# Surgery for long tubular intestinal duplication with massive hemorrhage: a case report and literature review

**DOI:** 10.1186/s40792-024-01829-6

**Published:** 2024-02-02

**Authors:** Chiyoshi Toyama, Hiroto Mizushima, Kenichi Kinjo, Yohei Masunaga, Yasuko Fujisawa, Isao Miyairi, Yukichi Tanahashi, Satoshi Osawa, Toshio Sawai

**Affiliations:** 1 Department of Pediatric Surgery, Hamamatsu University School of Medicine, University Hospital, Handayama, 1-20-1, Hamamatsu, Shizuoka 431-3192 Japan; 2 Department of Pediatrics, Hamamatsu University School of Medicine, University Hospital, Handayama, 1-20-1, Hamamatsu, Shizuoka 431-3192 Japan; 3 Department of Radiology, Hamamatsu University School of Medicine, University Hospital, 1-20-1, HandayamaHamamatsu, Shizuoka, 431-3192 Japan; 4 Department of Endoscopic and Photodynamic Medicine, Hamamatsu University School of Medicine, University Hospital, 1-20-1, HandayamaHamamatsu, Shizuoka, 431-3192 Japan

**Keywords:** Duplication, Tubular, Melena, Wrenn, Gastric mucosa

## Abstract

**Background:**

Long tubular duplication is a rare congenital intestinal disease, that can lead to emergency situations marked by massive hemorrhage. However, preoperative diagnosis and surgical treatment are challenging. This report presents preoperative images and details a surgical procedure for long tubular intestinal duplications with massive hemorrhage.

**Case presentation:**

A 3-year-old boy presented to the emergency department with melena. Despite undergoing a Tc-99m pertechnetate scintigraphy one year prior, which revealed nonspecific findings with enhancement of some parts of the intestine, enhanced abdominal CT revealed an edematous small intestine with luminal extravasation. The patient received a transfusion of red blood cells; however, his hemoglobin level did not improve. Arterial angiography and double-balloon endoscopy revealed no remarkable findings. Exploratory laparotomy revealed a long tubular duplication in half of the small intestine. Utilizing the Wrenn procedure, we successfully removed all duplicate mucosa. Pathological findings showed that almost all duplications contained gastric mucosa and revealed an ulcer with a ruptured arterial vessel. His symptoms were resolved, and the hemoglobin level stabilized. At 2 months postoperatively, no surgical complications were present.

**Conclusions:**

Effective management of long tubular duplications with massive hemorrhage involves timely application of the Wrenn procedure. Recognition of specific imaging findings is crucial to prompt exploratory laparotomy, ensuring optimal outcomes and preventing delays in treatment.

## Background

Duplication occurs in one out of every 10,000 live births, with approximately 80% of patients exhibiting nonspecific symptoms before the age of 2 [1]. These duplications are classified as cystic (80%) or tubular (20%), with the small intestine being the most common gastrointestinal site. Common symptoms include vomiting, abdominal pain, and palpable masses. Gastrointestinal hemorrhage may occur in duplications containing ectopic gastric mucosa [2]. While rare, a long tubular intestinal duplication with intestinal hemorrhage can be fatal, and its diagnosis and treatment remain challenging.

Preoperative diagnosis of duplication is often difficult and is frequently performed during surgery [3]. While imaging examinations are generally considered ineffective, duplications with massive hemorrhage necessitate an emergency diagnostic workup. Imaging investigations in pediatric patients with melena include enhanced computed tomography (CT), endoscopies, and Tc-99m pertechnetate scintigraphy. However, comprehensive findings regarding long tubular intestinal duplications are not well established.

Treatment for duplications typically involves removing the duplications entirely. The surgical management of long tubular duplications pose a challenge [4], as injury to the mesenteric vessels can result in short bowel syndrome. Various procedures for duplication have been reported, including Bianchi and Wrenn, and the creation of large windows (marsupialization) between the duplication and the adjacent intestine [5–9]. Although surgery for long tubular intestinal duplications remains controversial, there are few reports summarizing the surgical treatment for this disease. In this report, we present the preoperative images and the surgical procedure for a case of long tubular intestinal duplication with massive hemorrhage.

## Case presentation

**Fig. 1:**
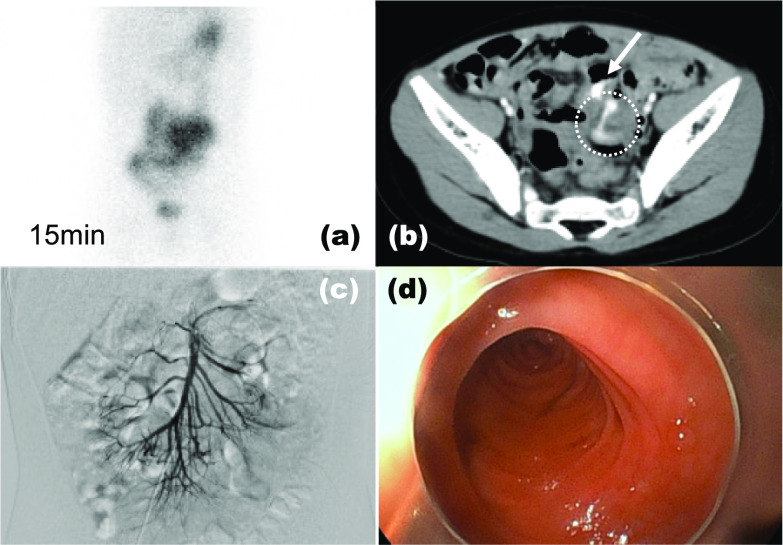
Preoperative images: **a** Tc-99m pertechnetate scintigraph. A part of the intestine is diffusely contrasted. **b** Enhanced CT showing extravasation into the small intestine (arrow) and the hypervascular lesion (dotted circle). **c** Arterial angiography indicating no remarkable extravasation. **d** Tran-sanal double-balloon enteroscopy showing no obvious abnormality on the mucosal side from the Bauhin valve up to 100 cm into the small intestine

**Fig. 2:**
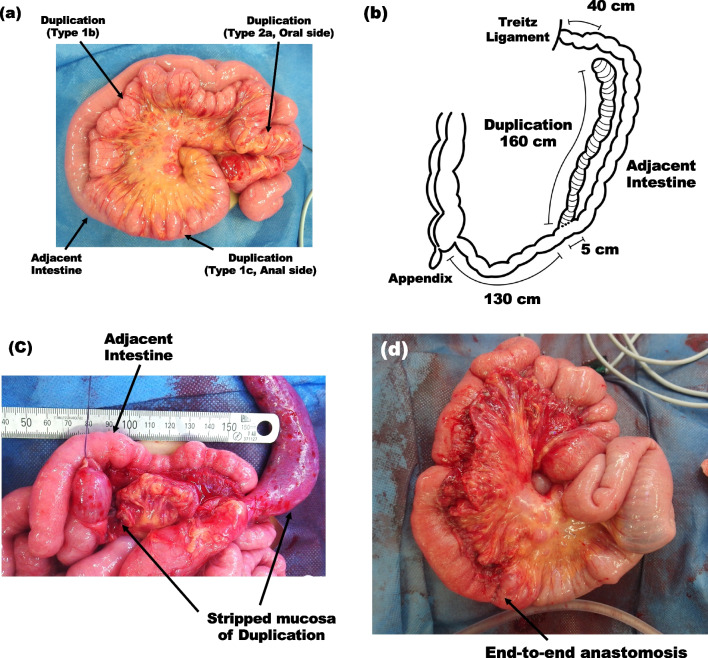
Operative findings: **a** the long tubular duplication located beside the intestine (160 cm). The classifications in the relation to the mesenteric vessels are primarily type 1b, with some type 1c and type 2a. **b** The schema of the duplication and intestine. The duplication is adjacent to a 40- to 200-cm portion of the small intestine and connected at one end. **c** Wrenn procedure, in which the mucosa of the duplication was stripped. **d** The mucosa of type 1b and type 2a duplications were resected using the Wrenn procedure (mucosal stripping of the duplication) and the type 1c duplication was resected with the adjacent intestine (5 cm) and end-to-end anastomosis

**Fig. 3:**
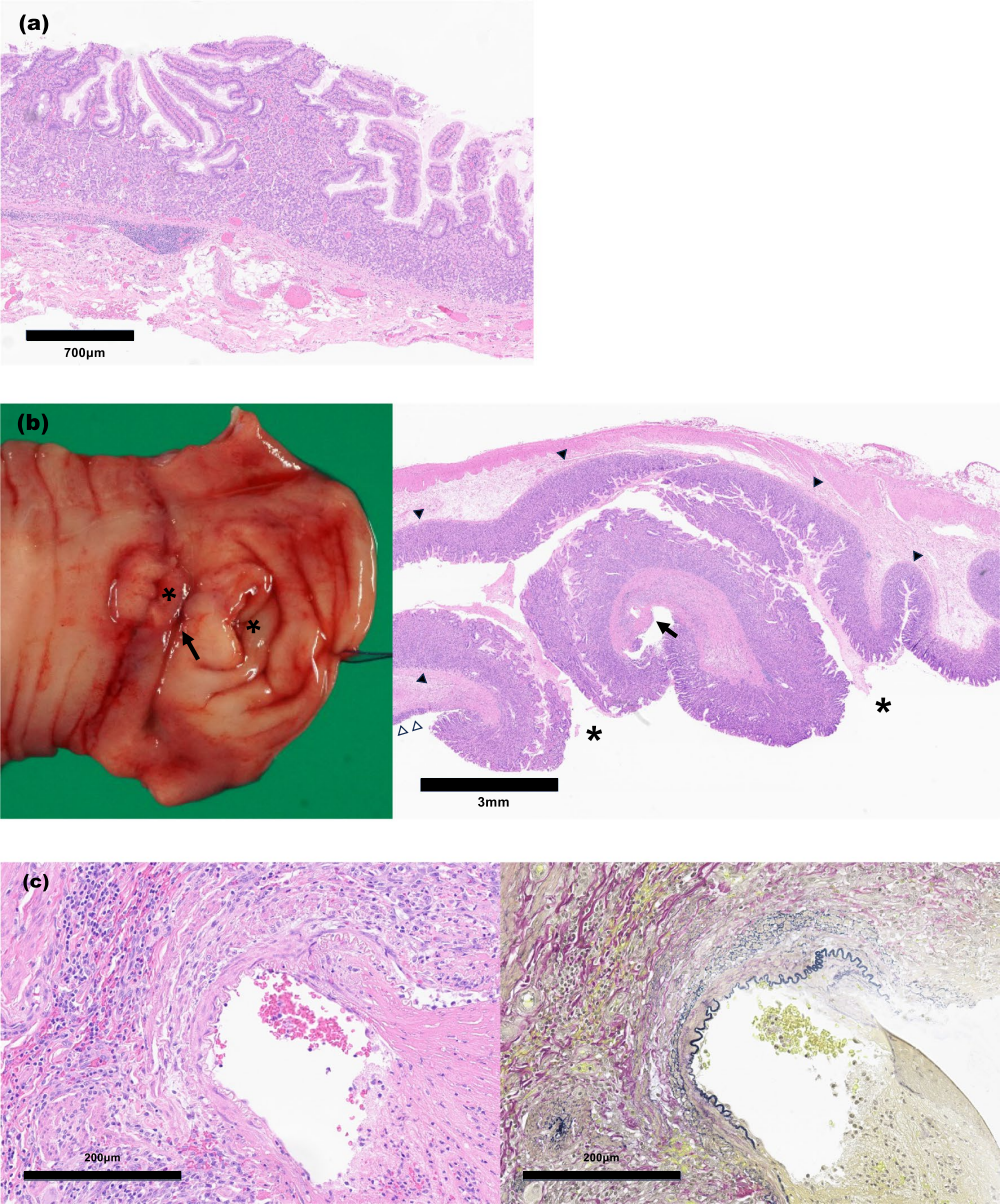
The pathological findings from the duplications. **a** The type 1b and type 2a duplications. Almost all the duplications include gastric mucosa. The duplication is on the dissected submucosal layer. **b** The macroscopic (left) and microscopic (right) findings of the anal side of the duplication. The duplication connects to the adjacent intestine via two orifices (asterisks). An ulcer is located in the center of the orifices (arrow). Most of the duplication contains gastric mucosa (black triangle) while the adjacent intestine has normal mucosa (white triangle). **c** The ruptured arterial vessel in the ulcer (high-power field). The ruptured vessel opens to the lumen of the intestine with fibrin clots (left, hematoxylin–eosin staining). Elastic Van Gieson staining showing that the ruptured vessel contains elastic fibers as an arterial vessel (right)

A 3-year-old boy presented to the emergency department with an episode of melena and black tarry stools. He had experienced similar symptoms a year prior and had undergone several examinations, including colonoscopy and Tc-99m pertechnetate scintigraphy (Fig. 1a). The colonoscopy findings were normal, and scintigraphy revealed nonspecific findings with some parts of the small intestine enhanced but not spotted. We suspected it was a small intestinal hemorrhage. The patient was discharged from the hospital and scheduled to undergo esophagogastroscopy and capsule endoscopy to identify the root cause. After readmission to the emergency department, his blood analysis revealed a hemoglobin level of 9.6 mg/dL (from 13 mg/dL), leukocyte count of 9.2 × 10^9^/L, and creatinine level of 0.28 mg/dL, and a urea nitrogen level of 20.7 mg/dL. Enhanced abdominal CT revealed an edematous small intestine with extravasation into the lumen of the small intestine (Fig. 1b). Although angiography via the femoral artery was performed immediately and the hypervascular lesion was detected in the area consistent with that on the enhanced CT, the extravasation resolved by that time (Fig. 1c). The patient received an overnight transfusion of 240 mL of red blood cells (RBCs), but his hemoglobin level did not increase. We performed trans-anal double-balloon enteroscopy under general anesthesia because the hypervascular lesion was located in the ileum on the enhanced CT. Although no specific findings were observed from the Bauhin valve to the 100 cm ileum on the mucosal side, the poor operability of the endoscope indicated that an extramural disease could be present. Exploratory laparotomy was immediately performed through an umbilical incision. The tubular duplication was adjacent to a 40- to 200-cm portion of the small intestine and connected at one end (Fig. 2a). The relation between the duplication and the normal intestine and the length of the intestines were checked from both directions: from the Treitz ligament to terminal ileum and terminal ileum to the Treitz ligament, and the normal intestines without the duplication were 40 cm jejunum and 130 cm ileum (Fig. 2b). The classifications of the relationship to the mesenteric vessels and duplication were mostly type 1b, with some types 1c and 2a [10]. We first encircled the mucosa of the center of the type 1b duplication and dissected the oral side with incisions to the mesentery and the muscular layer of the duplication (Fig. 2c). The mucosa of the 2a duplications (10 cm) was cored from the same incision without mesenteric injury. The remaining intestine was dissected along the anal side. For the type 1c duplication, we resected the duplication with the adjacent intestine (5 cm) and performed end-to-end anastomosis using simple interrupted sutures because it was challenging to preserve the adjacent intestine (Fig. 2d). The operative time was 6 h (without endoscopy), and there was 200 mL of additional blood loss. The transfusions included RBCs (150 mL) and fresh frozen plasma (FFP; 140 mL). The pathological findings indicated that most duplications involved the gastric mucosa (Fig. 3a, b). The type 1b duplication site was dissected on the submucosal layer (Fig. 3a). The anal side of the duplication was connected to the adjacent intestine, and an ulcer was located at the center of the two orifices (Fig. 3b). The ulcer contained a ruptured arterial vessel, similar to a Dieulafoy ulcer (Fig. 3c). The patient was extubated on postoperative day (POD) 0, and normal feeding was started on POD 4. The hemoglobin level remained stable at 13 mg/dL. No surgical complications were observed, and the patient was discharged on POD 7. Two months postoperatively, follow-up found that the patient had no symptoms or complications.

## Discussion

Surgery for long tubular duplications remains challenging. We performed emergency surgery for a long tubular duplication with massive hemorrhage, precisely resecting the duplicated mucosa while adequately preserving the adjacent intestinal tract. Previously documented cases of long tubular intestinal duplications are shown in Table 1 [4, 6, 8, 11–13]. Most patients presented with melena, and their duplications contained gastric mucosa. In our case, a significant portion of the duplication involved the gastric mucosa.

**Table 1:** Reported cases of long tubular intestinal duplication

Author(year)	Age(year)	Symptoms	Tc-99m scintigraphy	Lengths (cm)	The presence of gastric mucosa	Procedure	Complication	Outcome
D L Schwartz(1980)	0	Melena	NA	102	Yes	NA	No	Alive
R. W. Norris(1986)	0	Melena	NA	130	NA	NA	No	Alive
M Sham(2010)	2	Abdominal pain	NA	120	No	Bianchi	No	Alive
A Bhattacharya(2013)	6	Fever Abdominal pain Melena	Positive	150	Yes	Wrenn	Sepsis	Death
V Khanna(2018)	0	Abdominal mass	Positive	90	Yes	Staged Surgery(Wrenn)	No	Alive
P G Vaze(2023)	9	Melena	Positive	80	Yes	Wrenn	Re-operation	Alive
Our case	3	Melena	Positive	160	Yes	Wrenn	No	Alive

*NA* not available

Diagnosing tubular duplications is challenging. The use of Tc-99m pertechnetate scintigraphy for duplication diagnosis was reported by Bhattacharya et al. and Kumar et al. [13, 14]. They suggested that the 99mTc pertechnetate could concentrate in a duplication cyst, similar to Meckel's diverticulum, offering a potentially useful diagnostic tool. In our case, Tc-99m scintigraphy showed widespread uptake that may have indicated long duplication, but it was difficult to differentiate from nonspecific uptake because it was not a spot uptake. Although the safety and usefulness of double-balloon enteroscopy have been reported in recent years [15], we could not visualize the target lesion using tran-sanal double-balloon enteroscopy because of the difficulty in inserting the scope due to the presence of a long duplication outside the small intestine. While capsule endoscopy could be beneficial for excluding other diseases by examining the entire small intestine, its ability to diagnose long duplication outside the small intestine remains uncertain. Consequently, laparoscopic diagnosis might have been the most valuable and necessary diagnostic method in this particular case. Interestingly, most reported cases were positive on Tc-99m scintigraphy, and their duplications contained gastric mucosa (Table 1). Although not specific to the syndrome, it is worth noting that this syndrome often presents with such imaging findings. When preoperative imaging does not provide sufficient information for a definitive diagnosis, ultrasonography, upper gastrointestinal examination, or magnetic resource imaging might provide additional information that can help with the diagnosis of this disease.

Surgery for long tubular duplications of the gastric mucosa presents certain challenges. Although gastric mucosa in duplications should be removed due to malignancy potential and hemorrhage, removal of the duplication without injury to the adjacent intestine or its feeding vessels is not easy because duplications are typically located in mesentery [11]. In our case, we successfully performed the Wrenn procedure, wherein the duplication was dissected on the submucosal layer to ensure complete removal of all mucosa (Fig. 3a) [9]. In our review, most patients were treated using the Wrenn procedure to avoid short bowel syndrome. Thus, the Wrenn procedure may be safe and effective for long tubular intestinal duplications. However, the optimal choice of procedure may depend on the relationship between the duplication and the mesenteric vessels or adjacent intestines, emphasizing the need for a tailored approach in each case.

In the differential diagnosis of pediatric patients with massive hemorrhage of the small intestine, due consideration should be given to the possibility of long tubular intestinal duplications. When this condition is suspected based on imaging findings, caution should be exercised against the indiscriminate performance of exploratory laparotomy.

## Conclusions

Adequate implementation surgical procedures, particularly employing the Wrenn technique, proves effective in managing patients with long tubular duplications and massive hemorrhage. Timely investigation of this condition, guided by thorough knowledge of imaging findings, is paramount to preventing unnecessary delays associated with exploratory laparotomy.

## Data Availability

Not applicable.

## Abbreviations

CT: Computed tomography
RBC: Red blood cell
FFP: Fresh frozen plasma
POD: Postoperative day
DBE: Double-balloon endoscopy
NA: Not available
